# The Management of Brain Metastases—Systematic Review of Neurosurgical Aspects

**DOI:** 10.3390/cancers13071616

**Published:** 2021-03-31

**Authors:** Martin A. Proescholdt, Petra Schödel, Christian Doenitz, Tobias Pukrop, Julius Höhne, Nils Ole Schmidt, Karl-Michael Schebesch

**Affiliations:** 1 Department of Neurosurgery, University Hospital Regensburg, 93053 Regensburg, Germany; martin.proescholdt@ukr.de (M.A.P.); petra.schoedel@ukr.de (P.S.); christian.doenitz@ukr.de (C.D.); julius.hoehne@ukr.de (J.H.); nils-ole.schmidt@ukr.de (N.O.S.); 2 Wilhelm Sander Neuro-Oncology Unit, University Hospital Regensburg, 93053 Regensbur, Germany; tobias.pukrop@ukr.de; 3 Department of Medical Oncology, University Hospital Regensburg, 93053 Regensburg, Germany

**Keywords:** brain metastases, surgical resection, infiltration, neuronavigation, fluorescence-guided surgery

## Abstract

**Simple Summary:**

In this comprehensive review, we focused on the neurosurgical treatment as an integrative part of the challenging multidisciplinary management of cerebral metastases, a neuro-oncologic entity, which has been observed to have an increased incidence over the last years. In selected cases, the surgical removal of the space-occupying mass reduces the intracranial pressure, normalizes the metabolic environment, reduces the symptom burden, and allows for the intensification of local and systemic adjuvant treatment. In detail, we discuss the incidence of brain metastases, the role of surgical resection, as well as the evolution of current neurosurgical techniques, the surgical morbidity and mortality of single and multiple lesions, and we enlighten the role of surgery for recurrent tumors.

**Abstract:**

The multidisciplinary management of patients with brain metastases (BM) consists of surgical resection, different radiation treatment modalities, cytotoxic chemotherapy, and targeted molecular treatment. This review presents the current state of neurosurgical technology applied to achieve maximal resection with minimal morbidity as a treatment paradigm in patients with BM. In addition, we discuss the contribution of neurosurgical resection on functional outcome, advanced systemic treatment strategies, and enhanced understanding of the tumor biology.

## 1. Epidemiology of Brain Metastases

Cancer is the second most prevalent cause of death worldwide [1], with lung, breast, colorectal, and prostate being the most frequently affected organs [1]. Tumor cell seeding into the central nervous system [2], mostly localized in the brain parenchyma, the cerebrospinal fluid (CSF), the dura, and the bone structures of the skull, is a frequent complication of advanced cancer. In addition to reduced life span, brain metastases (BM) frequently cause focal neurological deficits, cognitive impairment, and significant life quality reduction [3]. By far, outnumbering primary brain tumors by about eight- to ten-fold, BM are the most frequent intraparenchymal tumors of the brain [4] and they show an increasing incidence [5]. There are three potential reasons for this epidemiological trend: (A) the improved imaging technology allows for detecting brain metastases earlier and on a higher frequency in cancer patients [6]. In particular, the high-resolution contrast-enhanced T1-weighted MR imaging in addition to fluid-attenuated inversion recovery (FLAIR) and diffusion-weighted imaging has significantly improved the sensitivity for BM detection [7]; (B) the more effective systemic treatment of systemic cancer, especially the clinical application of first- and second-generation tyrosine kinase inhibitors in addition to immune checkpoint inhibitors, has profoundly increased the life span to the affected patients in which BM can develop [8]; and, (C) the brain shows a restricted bioavailability to several antineoplastic drugs due to the blood-brain barrier (BBB), which is, in contrast to common belief, only heterogeneously altered in BMs [9,10]. Another essential feature of the BBB is the restriction of cellular and humoral immune surveillance leading to a relative immune-privileged status of the brain [11]. This may cause the central nervous system to develop into a refuge site in which metastatic cancer cells are protected from immune–mediated attacks and destruction [12]. Finally, the specific biochemical environment that is regulated by the BBB appears to foster the seeding and proliferation of specific tumor cells, in particular clones with neuroepithelial differentiation, like small cell cancer or melanoma cells [13,14]. The summation of these aspects promotes the development of metastases in the brain as a pharmacological sanctuary compartment, despite successfully controlling the systemic disease [15].

## 2. The Incidence of Brain Metastases

Although it is challenging to define the exact frequency of BM occurring in cancer patients due to the different data sources ranging from autopsy series [16,17] through observational studies [18] to epidemiological reports [19,20,21,22], approximately 20–40% of patients with cancer are affected which equals about 200,000–300,000 cases in the United States per year [23]. It has been consistently observed that BM consistently occur more frequently in specific primary cancers. Lung cancer has been reported to cause the highest number of BM cases with an incidence rate of 9–46%, depending on histological type, Epidermal Growth Factor Receptor (EGFR) mutation status, and disease stage [24,25]. Breast cancer carries a BM risk of 0.4–9.2%, with most of the cases occurring in Human Epidermal Growth Factor Receptor 2 (HER2)-positive or triple-negative patients [26,27]. Finally, malignant melanoma has the highest risk from all primary cancers to produce BM, even though most BM cases are related to lung cancer. The incidence rate of melanoma-associated BM is reported between 6.9% up to 18.5%, with a significant association to the male gender, young age, and the presence of extracranial metastases [21,22,28,29]. Although most of the BM patients present with singular or solitary lesions [30,31], cases with multiple metastases are frequent, especially in cases with unknown primary cancer [32].

The BM location is an essential factor defining the clinical symptoms, management strategies, and prognosis of the affected patients [33]. Although the frontal lobe has been reported to be the most frequent site within the brain [34], primary cancer type and specific biological features strongly influence the site preference of CNS spread. In particular, the posterior fossa’s metastatic tumors have been found to occur more frequently in patients with colon cancer [35] and HER2-positive breast cancer [36].

## 3. The Role of Surgical Resection in the Management of BM Patients

Traditionally, the prognosis of patients with BM has been considered to be extremely poor [37], with a median overall survival of about 1–2 months [38]. Palliative whole brain radiation was employed as standard therapy, extending the life span by about 2–4 months [39]. Palliative treatment also includes corticosteroids, commonly dexamethasone, in a dosage of 6 mg every 6 h, especially if the patients show neurological impairment due to perifocal edema [40]. Anti-convulsive therapy is indicated for patients presenting with tumor–associated epileptic seizures. However, no prophylactic anti-seizure medication has been recommended [41]. Basic research focusing on the biology of brain metastases has generated a new armamentarium of treatment modalities, which significantly improved BM patients’ outlook, especially those with tumors harboring treatable molecular alterations [22,42,43,44]. In this multidisciplinary treatment matrix, microsurgical resection plays a vital role due to four reasons:

(1) The primary cause of CNS failure-related death in BM patients is the intracranial mass lesion that results in elevated intracranial pressure and increasing brain stem compression [45]. The resection of the intraaxial lesion, accompanied by the reduction of perifocal edema based on removing the leaky peritumoral vasculature, results in an improvement of intracranial compliance, reduced intracranial pressure, and improved overall survival. This aspect is highlighted in the two landmark studies prospectively demonstrating the prolonged median overall survival in BM patients receiving microsurgical resection plus whole-brain radiation therapy (WBRT) versus WBRT only [46,47]. In more recent, mostly retrospective studies, a significant effect of surgical resection on the functional condition and overall survival was demonstrated [48,49,50].
(2) The brain as a host organ is highly susceptible to functional impairment due to local pressure and changed local biochemical environment in the context of metastatic tumor growth [51]. Consequently, the surgical evacuation of a metastatic tumor, mainly if located in an eloquent area of the brain, will frequently lead to reduced symptom burden and the improvement of focal neurological deficits [52,53,54,55]. In a recent publication reporting functional improvement rates in BM patients, it was demonstrated that more than 20% of all BM patients suffer from hemiparesis, 11.3% display speech disturbances, and 23.2% show signs of cerebellar dysfunction. That portfolio of focal neurological deficits has led to a reduced functional independency in most of the affected patients, which was significantly improved after surgical resection [55]. Concordantly, a recent report highlighted the importance of neurological deficits on the overall prognosis in patients with BM [3]. Consequently, the impact of surgical resection on neurological function not only enhances the potential quality of life in these patients, but it also leads to an improved postsurgical Karnofsky Score (KPI) and the recursive partitioning (RPA) score, which is an important parameter to tailor adjuvant treatment structure [56]. This effect is even more pronounced in elderly patients with symptomatic BM undergoing surgical resection [57]. An improvement of KPI and RPA score in this prognostically poor patient subgroup was associated with a much higher likeliness to receive adjuvant local and systemic treatment, including molecular targeted therapy, resulting in more prolonged overall survival [57].
(3) As shown in Patchell`s landmark paper, even in patients that were diagnosed with metastatic cancer, an intraaxial lesion is not a metastatic tumor in 11% of the affected patients [47]. One might hypothesize that the application of modern imaging technologies might have improved the diagnostic sensitivity and specificity of the current diagnostic platforms [58,59,60]. However, even high-end imaging approaches, such as amide proton transfer-weighted imaging, molecular MRI [61,62,63], or positron emission tomography [64], do not allow for the definitive diagnosis of an intraaxial lesion, in a patient with metastatic cancer. That indicates the pivotal need for histological confirmation of suspicious lesions, which is well reflected by the clinical experience of treating neurooncologists [65].
(4) Finally, increasing evidence has indicated significant differences in the biology of primary cancers and the corresponding BMs, possibly resulting in additional therapeutic options [66]. In a practice-changing study, Brastianos et al. have demonstrated that more than 50% of all analyzed BMs show treatable molecular alterations that were not detectable in the primary tumor [67]. The potential reason for this observation might be the brain’s specific microenvironment, which induces profound changes in the biology of those cancer cells, which managed to home in the CNS. The extracellular matrix of the brain and the specific metabolic conditions of the CNS may prompt the cancer cells to acquire a more brain-specific phenotype [68,69]. For example, a recent study has demonstrated a significant induction of HER-2 protein expression in the BM tissue of metastatic breast cancer patients as compared to the primary tumor, potentially leading to a successful treatment strategy with anti HER-2 substances [70]. Consequently, a microsurgical resection may serve the purpose of tissue acquisition for molecular analysis, leading to so far undetected targets for systemic treatment, improving the prognosis of the affected patient population.

## 4. Surgical Morbidity and Mortality in the Resection of Brain Metastases

When analyzing the frequency of surgical complications, there appears to be a positive trend along the time axis [71]. After a highly concerning surgical morbidity rate in BM patients of 24.8% was reported in 1972 [72], this rate has significantly dropped to 2–10% in more modern series [46,47,55,57,71,72,73,74,75,76]. In particular, the most frequent complications are postsurgical hemorrhage (2.7%), pulmonary embolism (2.2%), CSF leakage (0.8%), and stroke (0.6%) [55]. As a risk factor for higher surgical morbidity, age has been considered to be an important parameter [77]. However, a recent study analyzing 805 BM patients that were stratified by an age threshold of 65 years did not find a significantly higher morbidity rate in the elderly patient strata [57]. This argues against withholding surgical treatment strictly based on age [78]. The same applies for comorbidities, which were expected to correlate with surgical morbidity [79]. In the above mentioned study, the Charlson comorbidity score [80] was, as expected, higher in the elderly subgroup; however, there was no correlation between this score and the occurrence of surgical complications [57]. The development of a permanent neurological deficit following surgical resection is the greatest concern regarding surgical morbidity in BM patients, since this has been shown to negatively influence the overall outcome [81]. Despite limited comparability due to heterogeneous study designs, the frequency of surgery related permanent neurological worsening range from about 6% [50,54,82] up to 11% [52,53]. Clearly, the higher neurological deficit rates are associated with tumors in eloquent areas. For these tumors, a highly sophisticated technical portfolio consisting of functional imaging [83], DTI-based tractography [84], navigated transcranial magnetic stimulation [85], as well as awake craniotomy [86] with direct cortical and subcortical stimulation is available, to keep the surgery induced impairment at a minimum level. Interestingly, the frequency of neurological complications appears to be significantly influenced by the specific neurosurgical methodology, in particular whether the resection was performed en bloc versus in the piecemeal technique [87]. With regard to surgical mortality that is defined by the death of a patient within 30 days of surgery, there again appears to be an improvement from the historical 8–11% [46,72,73] down to 2–4% in the more recent studies [55,57,76,88]. Presumably, the improved multidisciplinary perioperative management of BM patients consisting of corticosteroids plus gastrointestinal prophylaxis, the application of antiepileptic drugs if required, and prophylactic anticoagulation has greatly contributed to a reduced surgical mortality in BM patients [89,90].

## 5. Resection of Multiple Brain Metastases

Based on clinical studies, between 30–50% of all BM patients present with multiple lesions, depending on the primary cancer type [31,91,92,93]. Several studies demonstrated that the occurrence of multiple metastatic tumors indicate a poorer prognosis when compared to singular or solitary lesions [29,94,95,96]. In contrast to single BMs, in which the beneficial role of surgical resection has been established by prospective trials [46,47], no class I evidence exists for the patient population with multiple BM. Bindal et al. have demonstrated that, if all lesions are removed, the survival outcome in patients with multiple BM is no longer inferior to patients with single lesions [74], a finding that was confirmed by more recent studies [97,98]. However, the importance of removing all lesions with regard to overall survival outcome seems to depend on the primary tumor type. In a study that was recently performed in 51 patients with multiple BM from NSCLC (non-small cell lung cancer), no difference was found whether all lesions removed or not, provided that the residual tumors were treated with radio- and chemotherapy [99]. Interestingly, the study by Salvati et al. demonstrated a neurological improvement rate in patients after resection of multiple lesions, which was similar to patients presenting with single lesions [98]. Similarly, Schackert et al. demonstrated comparable KPI improvement rates in patients with multiple or single lesions [100] and, although the presence of multiple lesions was associated with a poorer overall survival, the most prominent prognostic factor was the postsurgical KPI, regardless of the number of lesions. This is in accordance with our results regarding the improvement of neurological function and KPI, which was similar between patients with single and multiple lesions [55]. In conclusion, although no class I evidence is available, surgical resection in patients with multiple lesions can reduce neurological symptom burden and improve functional independence. In the context with modern adjuvant treatment, including targeted therapy or immune checkpoint inhibition, the removal of a large metastatic mass leading to decompression of the brain and opening a time window for augmented postsurgical treatment may be an adequate strategy in selected BM patients [101].

## 6. The Role of Surgery for Recurrent Brain Metastases

Metastatic tumors of the brain were traditionally considered to be well-delineated with very limited infiltration of the surrounding tissue [102]. Careful histological studies have revised this assumption [103,104], which is corroborated by a significant local recurrence rate after both surgical resection [47] and focal radiotherapy [105]. The improved systemic disease control rates due to modern treatment strategies [8] lead to an increased number of cases with recurrent BM requiring salvage therapy [106]. Surgical re-resection is a valid option in selected patients with recurrent BM, according to a recent review [107]. Unfortunately, there are only retrospective case-series available to establish the beneficial impact of surgery in this setting [107,108,109,110,111,112,113,114,115]. An indication for re-operation was reported in several studies if patients show a rapidly progressing, symptomatic mass lesion that was surgically accessible and at the same time display controlled systemic disease and a good functional condition reflected by a KPI score of >60 [106,112,114,116,117,118]. The median OS after salvage operation ranged between 7.5 months [114] and 20.2 months [106], and depended on presurgical performance status [113], time between initial and salvage BM surgery [109], as well as extent of resection during re-operation [114]. Bindal et al. have summarized all of the potential prognostic factors in a grading system to predict outcome after salvage surgery, including the status of systemic disease, preoperative KPI score, time to recurrence, age, and primary tumor [111]. Consequently, the median survival rates after re-resection of recurrent BM ranged from 13.4 to 3.4 months, depending on the grading score [111]. Interestingly, five retrospective studies reported functional improvement rates in patients with symptomatic BM recurrence between 62–90% after surgical resection [106,109,111,115,117], highlighting the beneficial impact of surgery on symptom burden and functional independency. However, the management of recurrent cerebral metastases is challenging, as the majority has already been treated with radio- and chemotherapy, potentially rendering any cranial re-operation difficult in terms of an increased risk of wound healing disorders, infections, hemorrhages, and CSF-fistulas due to scarring, arachnoiditis, and pathological dural adherences of edematous brain tissue [106,117,119]. The morbidity rates reported in the available studies range from 31% [110] to 0% [106,109,112], and they may depend on the specific status of the patients recruited for the individual studies. Despite the high degree of heterogeneity between the studies, no significantly higher morbidity rate can be concluded between the studies reporting initial [46,47,55,72,73,76] and salvage resection [109,110,111,112,115,116,117] for BM. The same assumption seems to apply to surgical mortality of salvage surgery for BM, which was reported to be between 0% [109,110,111,113,115] and 3.1% [117], and it does not profoundly differ from the mortality rates observed after initial BM resection [46,47,50,53,55,57,73,88]. Taken together, in patients showing a KPI > 60 and a large, symptomatic recurrent metastatic mass, which is surgically accessible, re-resection can provide symptomatic relief and contribute to improved functional independency with acceptable morbidity and mortality rates.

## 7. Evolution of the Surgical Techniques

Basically, the neurosurgical treatment modalities’ refinements were achieved by the implementation of various technologic advances, resulting in a better anatomical and physiological understanding of the affected brain. The identification of eloquent cortical areas is now routinely performed by functional magnetic resonance imaging (fMRI) in clinical practice [83,120], which can be complemented by navigated transcranial magnetic stimulation [85]. Furthermore, the presurgical identification of fiber tracts potentially displaced by the metastatic tumor, such as the pyramidal tract, or the uncinate fasciculus is crucial for determining an adequate surgical trajectory [84]. Additionally, intraoperative electrophysiological monitoring utilizing motor and sensory evoked potentials have been shown to help the attempt to radically resect the tumor and preserve brain function during microsurgical procedures in selected BM patients [121]. Finally, if the tumor location indicates high risks of postsurgical deterioration due to the location adjacent to eloquent brain areas, awake craniotomy with intraoperative testing and direct cortical and subcortical stimulation has been applied successfully [86].

**Figure 1:**
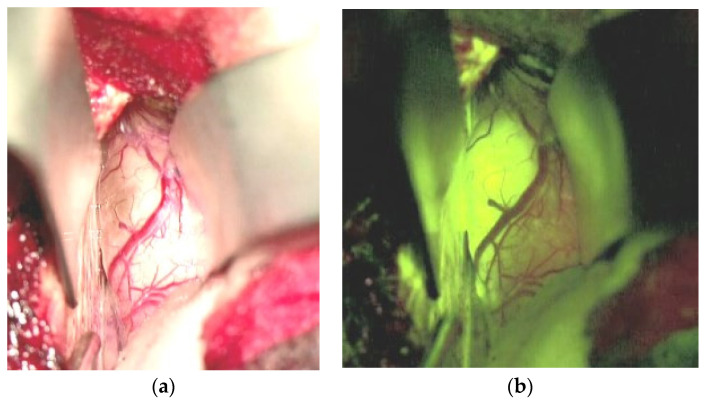
Renal cell cancer metastasis of the right cingulate gyrus, interhemispheric approach. (**a**): Surgical field under white light, (**b**): Surgical field under YELLOW 560 nm filter.

Significant efforts have been put forward to maximize tumor resection, as recent reports have shown a correlation between median overall survival in BM patients and the extent of resection as indicated by early postoperative MRI [76,122,123]. This important development is the intraoperative MRI, allowing for the control of resection quality while still in the procedure [124]. The development of fluorescence tracers for glioma surgery has now been applied and investigated in BM’s surgical management. After the significant improvement of resection quality in high-grade gliomas [125] with the application of 5-aminolaevulinic acid (5-ALA), this approach has been translated to the resection of BMs. In brief, 5-ALA is a precursor of hemoglobin synthesis, which explicitly induces the accumulation of protoporphyrin IX in tumor cells [126]. The tumor cells can be detected with high specificity and sensitivity in high-grade gliomas by applying blue fluorescent illumination. Unfortunately, in contrast to high-grade gliomas, which show 5-ALA induced fluorescence in almost all cases [127], only 48.5% of metastatic tumors do so [128]. Additionally, histological analysis of biopsies retrieved from residually fluorescent areas during BM resection showed false positive results (i.e., no tumor cell detection in fluorescent tissue) in about two-thirds of the cases [129]. In contrast, fluorescein sodium, a leakage tracer that accumulates under the circumstance of a disrupted blood-brain barrier (BBB), has a sensitivity of 94% in BM surgery and, therefore, may be an appropriate tool for resection quality improvement [130,131]. Figure 1 shows an interhemispherically approached renal cell cancer metastasis in the right cingulate gyrus under white light (Figure 1a) and under the YELLOW 560 nm filter illumination (Figure 1b).

**Figure 2:**
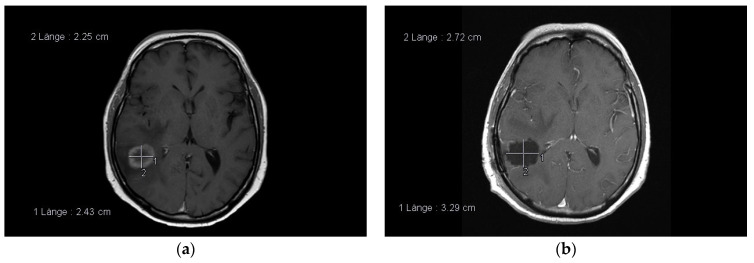
(**a**,**b**)**:** Examples of circumferential stripping. Adenocarcinoma metastasis in the right temporal lobe, note the resection cavity diameter exceeds the diameter of the contrast-enhanced lesion (pre- and postoperative. MRI, T1, contrast-enhanced).

A rather clear-cut border zone between the tumor and unaffected brain was easily identified under the filtered light, and tumor resection was continued under the YELLOW illumination. The fluorescence-guided technique in removing BM has been evaluated repeatedly concerning safety, feasibility, specificity, and radiographic outcome [130,131,132,133]. All of the authors concluded that this technique is significantly superior to resection under white light; however, prospectively designed clinical trials are still mandatory for verifying the clinical efficacy. In particular, one aspect needs further evaluation, namely the potential false positive signals due to BBB—alteration of the normal brain in BM patients who received focal or whole brain radiation therapy previous to the surgical procedure [134]. Especially in larger tumors beyond a diameter of 4 cm, an initial internal decompression with subsequent dissection of the tumor borders, frequently called “piecemeal“ resection, has been put forward. However, in contrast to an “en bloc“ resection, which essentially implies the dissection of the entire tumor out of its environment in one piece, the piece meal technique has been shown to induce a higher frequency of local recurrence [135] and leptomeningeal spread of metastatic tumors [136]. Therefore, it should be avoided if ever possible. The local control rates after surgical resection is another aspect of concern. In another landmark paper, Patchell has reported 46% recurrence at the BM’s original site in the resection group versus only 10% of the patients receiving postsurgical WBRT [137]. These results were confirmed by a more recent study by Nieder et al., who reviewed ten clinical trials and a total of 643 patients, and reported a local recurrence rate of 40% in the resection only versus 12% in the resection followed by radiation treatment group [138]. The best explanation for this observation is the presence of malignant cells left behind, despite gross total resection (GTR) based on intraoperative assessment and postsurgical MRI. Historically, BM were considered to be non-invasive tumors, with little to no infiltration of the peritumoral normal brain [102]. Several reports have challenged this view, revealing the presence of infiltrating tumor cells based on histological analysis [103,104,139]. Although a recent report of a prospective study performing biopsies in the peritumoral areas in 12 BM patients after GTR failed to demonstrate infiltrating tumor cells [140], another trial has revealed the presence of tumor cell infiltration in the adjacent brain parenchyma in 34.7% of all biopsies [141]. The central hypothesis explaining these conflicting results is the difference in the surgical technique utilizing either a Sudan-Nashold needle (negative trial) or tumor alligator forceps (positive trial), resulting in different sample volumes [142]. When considering the most recent evidence, a much higher degree of infiltration in BM needs to be assumed and incorporated into neurosurgical practice. One approach put forward by Yoo et al. [143] is a resection beyond the defined tumor borders, termed circumferential stripping [144] or total microscopic resection (MTR) in contrast to GTR (see Figure 2a,b). The study by Yoo has shown a reduction of two-year local recurrence rate by more than 50% in the MTR group when compared to the GTR group. The fact that the median overall survival was not different in the two groups can be explained by the circumstance that most BM patients succumb to the progress of the extracranial disease, rather than to CNS failure [145], therefore reducing the potential impact of MTR on overall survival. Significant concerns regarding supramarginal resection were raised regarding potentially higher surgery-induced morbidity rates due to the extended resection of adjacent brain parenchyma. However, Kamp et al. [146] have shown this approach’s feasibility without additional surgical morbidity, even in metastatic tumors located in eloquent brain areas.

**Figure 3:**
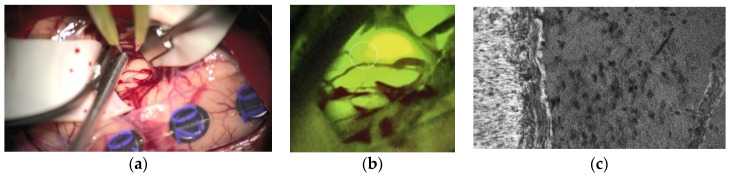
Lung cancer brain metastases (BM) of the right pre-motor cortex under white light (**a**), under YELLOW 560 nm illumination (**b**), and the brain-tumor-interface with CLE (**c**). The white circle in (**b**) indicates the point of the CLE image.

Finally, the modern era’s neurosurgeons may be called in to foster the understanding of BM infiltration by a more sophisticated tissue sampling out of the infiltration areas, which will allow the more precise analysis of biologically relevant signal pathways responsible for BM infiltration. Image-based identification of areas that are critical for this question [147], transferred into the routinely applied neuronavigation system [148] and potentially combined with novel intraoperative microscopic detection devices [149], may enable the approach of ’molecular biopsies´, in collaboration with basic science focusing on cell and molecular biology of BM infiltration to achieve a significantly improved understanding of the fundamental mechanisms that are related to this important aspect. Besides Raman spectroscopy [150,151], fluorescence-guided confocal laser-endomicroscopy (CLE) with Fluorescein sodium has shown promising results [152]. In BM, particularly, the in-vivo identification of the brain-tumor-interface is of utmost importance for non-residual tumor removal. Furthermore, in vivo CLE enables the real-time visualization of a living system, and significant improvements in understanding the pathophysiological coherences in BM can be expected within the next years. Figure 3a–c illustrate the surgical field finding under white light (Figure 3a), under YELLOW illumination (Figure 3b), and with CLE after frontal craniotomy for removing a lung cancer metastasis of the pre-motor cortex. The CLE image revealed the clear-cut brain-tumor interface with a high number of condensed tumor cells inside the BM.

## 8. Local Therapeutic Approaches Alternative to Surgery

Despite the significant development of surgical technology reviewed in this article, the majority of BM patients are not considered to be adequate candidates for microsurgical resection, due to general condition, the level of comorbidities, as well as number and location of the metastatic lesions [8]. Therefore, it is mandatory to mention two alternative local treatment options for BM patients in this context:

### 8.1. Laser Interstitial Thermal Therapy (LITT)

LITT implies the minimally invasive, stereotactically guided application of photons by a fiberoptic laser to eradicate lesions within the brain [153,154]. Most frequently, LITT is used to ablate both primary and secondary brain tumors, radiation necrosis, or epileptic foci [155,156]. The laser induced energy excites intracellular molecules, which leads to thermal energy release and subsequent eradication of the targeted lesion [157]. Pioneered by Sugiyama et al. [158], LITT was not immediately adopted as a neurosurgical technique due to limitations in particular with regard to the precise control of the applied thermal energy resulting in considerable toxicity [159]. However, the development of MRI-based real time thermal imaging has prompted a renaissance of this method [160], with the specific expectation to reduce neurological morbidity and mortality using this approach [161,162]. The current evidence indicates a specific segment of BM patients benefiting the most from LITT: a. Patients presenting with significant comorbidities not allowing for a safe microsurgical removal of the metastatic mass via craniotomy [163] and b. patients who have exhausted radio-oncological options still requiring local therapy due to increasing mass effects [164,165,166]. With regard to the target lesions, there are also several characteristics making LITT a preferrable choice [167]: (a) Deep seated lesions, which are surgically inaccessible. (b) Spherical or oblong configuration without signs of diffuse brain infiltration [154]. (c) Lesions that do not border large vessels or CSF spaces, since these structures may function as a heat sink, preventing the successful application of LITT [155]. In addition, the size of the lesion needs to be taken into account since larger lesions (>60–70 cm^3^) treated by LITT may be associated with a higher likeliness of clinically relevant LITT-induced cerebral edema [155,166]. In addition, it is mandatory to create complete thermal coverage of the target lesion to achieve maximal tumor control. In one prospective multicenter trial investigating LITT in 42 BM patients, the local recurrence rate was 25% in patients with complete, in contrast to 62.5% after incomplete, ablation [166], indicating that multiple LITT applications may be required under certain circumstances to generate maximal effects [162]. One study comparing surgical resection with LITT in patients with radiation necrosis or tumor recurrence after radiosurgery for BM demonstrated that surgery is superior to LITT in the resolution of neurological symptoms, but it did not cause improved progression free and overall survival rates as compared to LITT [168]. With regard to safety of LITT, the most frequent complications of LITT were intracerebral hemorrhage occurring in 1–14.2% [164,166]; cerebral edema [155,166]; and, neurological deficits both transiently (8.8–35.5%) and permanently (2.2–7.1%) [156,164,166,169]. In conclusion, LITT is a highly useful technology, provided that it is applied to the adequate patient segment, harboring lesions to which LITT is a feasible treatment option.

### 8.2. Stereotactic Radiosurgery (SRS)

Being introduced by Lars Leksell in the 1950s [170], stereotactic radiosurgery is defined as the application of multiple radiation beams focused on a target lesion in a stereotactic setting providing submillimeter precision treatment [171]. Because of its efficacy that is reflected by durable local tumor control rates and low toxicity, SRS has become the standard of care in a segment of patients with BM [172]. When comparing the efficacy of surgical resection versus SRS, the current evidence reflects highly heterogeneous results. Although two trials have demonstrated superior overall survival rates in the patients that were treated with surgical resection [48,173], this was not confirmed by another trial [174]. While two trials failed to detect significant differences in local tumor control rates between groups treated with surgery and SRS [173,175], three other studies reported superior control rates in the SRS treated patients [176,177,178]. Interestingly, one study comparing SRS alone versus the combination of surgical resection and SRS showed the best local control rates in this context [179]. A recent phase III trial attempted to prospectively compare surgery and SRS in BM patients, but it was terminated prematurely due to poor recruitment rates. The results that were derived from the limited number of patients did not show any difference between the local control rates or overall survival [180]. With regard to neurological symptoms, one trial reported superior recovery rates of pre-existing hemiparesis after surgical resection, however also a higher incidence of postsurgical neurological deficits, despite the use of neurophysiological monitoring during resection [52]. In conclusion, BM patients with deep seated, surgically inaccessible and/or multiple lesions are prime candidates for SRS [181]. That applies if the targeted lesions do not require histological or molecular pathologic re-evaluation, do not exceed an axial diameter of 3 cm, and do not cause any obstruction of CSF pathways [182].

## 9. Conclusions

The surgical resection of a metastatic tumor reduces mass effects and the intracranial pressure, leading to prolonged overall survival. Besides, the decompression of eloquent areas of the brain and normalization of the metabolic microenvironment causes a reduction of symptom burden and improvement of focal neurological deficits, which is associated with intensified adjuvant local and systemic treatment contributing to enhanced survival. Finally, the acquisition of tissue during surgical resection allows for the confirmation of the histological diagnosis of a metastatic tumor and the detection of brain-specific molecular alterations, which may lead to additional therapeutic options in the multimodal treatment of BM patients.
